# Optimization of dried garlic physicochemical properties using a self-organizing map and the development of an artificial intelligence prediction model

**DOI:** 10.1038/s41598-025-87167-5

**Published:** 2025-01-24

**Authors:** Hany S. El-Mesery, Mohamed Qenawy, Mona Ali, Merit Rostom, Ahmed Elbeltagi, Ali Salem, Abdallah Elshawadfy Elwakeel

**Affiliations:** 1 School of Energy and Power Engineering, Jiangsu University, Zhenjiang, 212013 China; 2 Agricultural Engineering Research Institute, Agricultural Research Center, Dokki, Giza, 12611 Egypt; 3 Mechanical Engineering Department, Faculty of Energy Engineering, Aswan University, Aswan, 81528 Egypt; 4 Academy of Scientific Research and Technology, ASRT, Cairo, Egypt; 5 Agricultural Engineering Department, Faculty of Agriculture, Mansoura University, Mansoura, 35516 Egypt; 6 Civil Engineering Department, Faculty of Engineering, Minia University, Minia, 61111 Egypt; 7 Structural Diagnostics and Analysis Research Group, Faculty of Engineering and Information Technology, University of Pécs, Pécs, Hungary; 8 Agricultural Engineering Department, Faculty of Agriculture and Natural Resources, Aswan University, Aswan, Egypt

**Keywords:** Machine learning, Continuous dryers, Infrared drying, Physicochemical properties, Plant sciences, Solid Earth sciences

## Abstract

The experiments were conducted at different levels of infrared power, airflow, and temperature. The relationships between the input process factors and response factors’ physicochemical properties of dried garlic were optimized by a self-organizing map (SOM), and the model was developed using machine learning. Artificial Neural Network (ANN) with 99% predicting accuracy and Self-Organizing Maps (SOM) with 97% clustering accuracy were used to determine the quality characteristics of garlic. Specifically, five key areas were identified, and valuable insights were offered for optimizing garlic production and improving its overall quality. The (aw) values for the sample ranged from 0.43 to 0.48. The maximum vitamin C content was 0.112 mg/g, followed by an air temperature of 40 °C and 0.7 m/s air velocity under 1500 W/m². The total color change values increased with IR and higher air temperature but declined with higher air velocity. Also, the garlic’s flavor strength, allicin content, water activity, and vitamin C levels decreased as the IR and air temperature increased. The results demonstrated a significant impact of the independent parameters on the response parameters (*P* < 0.01). Interestingly, the machine learning predictions closely matched the test data sets, providing valuable insights for understanding and controlling the factors affecting garlic drying performances.

## Introduction

Scientifically known as Allium sativum, Garlic has been cultivated globally for centuries due to its unique culinary and medicinal properties^4^. It has gained significant recognition as a valuable condiment in everyday food preparation. Garlic is rich in valuable components like allicin, selenium (Se), germanium (Ge), and superoxide dismutase (SOD), which are bioactive substances with numerous benefits for human health. Allicin, in particular, possesses antioxidant and antibiotic properties. Regrettably, despite garlic’s multiple health benefits to humans, around 30% of the garlic crop is lost during storage due to microbial and respiratory spoilage^5^. Fresh garlic has a high moisture content (more than 75%), which promotes garlic clove sprouting and rotting during storage, resulting in a shorter shelf life. Thus, to extend garlic’s shelf life while maintaining its nutritional value, the moisture content of garlic must be reduced to 6%^6^. Dehydrating garlic cloves using convection drying methods, such as tray dryers, is time-consuming and requires substantial energy^1^. In convective drying, the outer part of the garlic primarily undergoes heating through convection, while the remaining parts are heated through conduction. This process removes moisture from the garlic surface, resulting in a moisture gradient that facilitates the movement of liquid towards the outer layer^2^. However, convective drying has several disadvantages, including lengthy drying times, potentially negative effects on sensory characteristics and nutritional properties, and the migration of solutes from the inside of the food to the surface, resulting in case hardening. As drying technology advances, newer and more sophisticated dryers have been designed to address the shortcomings of hot air dryers and enhance the quality of the dried products^7^. In recent years, infrared dryers have gained popularity and garnered increased attention in developing countries because of their affordability, simplicity, and low operational costs. Infrared drying involves heating wet materials using infrared radiation^3,8^.

Accurately predicting drying parameters is vital for enhancing process conditions, reducing energy usage, and maintaining stable product quality. Artificial neural networks (ANNs) have become essential for analyzing intricate relationships across various fields, particularly food processing. Artificial intelligence (AI) and its data-driven counterpart, machine learning (ML), are rapidly evolving disciplines with increasing applications in modeling, simulation, control, and optimization within the food processing industry^9^. This integration facilitates automated decision-making, reducing human errors and enhancing operational efficiency in food drying^10^. Moreover, AI models demonstrate proficiency in predicting drying times and analyzing energy usage patterns, thereby enabling optimization to minimize resource consumption while preserving product quality^11^. A model that leverages the predictive capabilities of ANNs to accurately forecast the drying process based on input variables like product thickness, cabin pressure, and drying time is crucial for saving time and energy^12,13^. Jafari et al.^14^ found that the ANN model outperformed mathematical modeling methods regarding productivity and accuracy when predicting variations in the moisture ratio of green bell peppers during hot air fluidized bed drying. Similarly, Nadian et al.^15^ developed an ANN model to forecast the color changes in sliced apples during hot air drying. In another study, Sarimeseli et al.^16^ applied an ANN to model the microwave-drying kinetics of thyme leaves. Zalpouri et al.^17^ investigated the onion puree of varying thicknesses, and it was dried using RWD and CD methods to evaluate drying kinetics, physicochemical qualities, and thermal analysis of the dried powder. The results showed an increase in the thickness of the puree from 2 to 6 mm, and drying time increased from 135 to 240 min for RWD and 510 to 660 min for CD. To predict accurate MR of onion puree using RWD and CD, MLF- ANN with a back-propagation algorithm was used. ANN was an effective method for predicting MR for both drying methods. The result showed that the ANN model with 12 and 18 neurons in the hidden layer could predict the MR, with a high R² value for RWD and CD, respectively. Khazaei et al.^18^ employed ANN to predict and control the grape drying procedure in a hot air dryer. Although there are many studies on drying various agricultural products, no research specifically focused on drying garlic slices using continuous infrared-assisted convective hot air drying and optimizing the process with ANN techniques about quality attributes. Zalpouri et al.^19^ observed that an artificial neural network (ANN) model predicted MR. The mathematical modeling showed that the Exponential two-term model had the highest R² and lowest RSME and SEE values. Moreover, MR was accurately predicted using MLF-ANN with a back-propagation algorithm, outperforming the mathematical model. Mass transfer calculated using Dincer and Dost analytical approach showed Deff and hm in the range of 1.980 × 10^− 9^ to 1.839 × 10^− 8^ m²/s and 1.881 × 10^− 6^ to 5.653 × 10^− 6^ m/s, respectively. Kalsi et al.^20^ investigated the drying of *Stevia rebaudiana* leaves in a convection dryer under different air temperatures to analyze the drying kinetics, Artificial Neural Networks (ANNs), and Adaptive Neuro-Fuzzy System (ANFIS) models to predict the drying kinetics of leaves. Further, dried leaf powders were analyzed for color properties, ascorbic acid and total phenol contents, antioxidant activity, water activity, water solubility index, hygroscopicity, density, bulk porosity, flowability indices, Carr index, and angle of repose. The results showed that the ANFIS model with R² of 0.9998, offers a more accurate forecast of the drying kinetics of leaves dried in a convective hot-air dryer in comparison to mathematical and ANN modeling. This study employs a continuous dryer to examine the impact of varying infrared intensities, airflow, as well as air temperature on the physicochemical properties of garlic powder, including (total color change, flavor strength, water activity, vitamin C, and allicin content) during infrared drying. The research also aims to develop a machine learning-based model to forecast the changes in the physicochemical quality of dried garlic powder. A novel approach for discovering how to optimize the conditions of the study is offered by self-organizing maps (SOM). Principal component analysis (PCA) is used to understand drying conditions and the relationship between the physicochemical quality of dried garlic powder.

## Materials and methods

### Sample preparation

Fresh garlic clove (*Allium sativum L*.) was obtained from the local shop and reserved in the refrigerator at 4 °C for further analysis. After a 2-hour stabilization period at ambient temperature, the samples were peeled and sliced into 6 mm thick pieces using an industrial food slicer before drying. To prevent nonenzymatic browning, the garlic samples were immersed in a 4% w/w Sodium metabisulfite (Na_2_O_5_S_2_) solution at 25 °C. Fresh samples of approximately 500 g were dehydrated until 0.05 g water/g dry basis of final moisture content was achieved using the infrared dryer.

### Conveyor-belt dryer

The garlic drying process used a hybrid conveyor belt dryer with an infrared heating system. The dryer, measuring 3 × 1.5 × 8 m in length, height, and weight, includes a conveyor belt system, feeder, hot air convection system, drying chamber, product collection area, and infrared heating system.

#### Drying chamber

The dryer was designed with a drying chamber divided into two equal sections, each measuring 0.8 × 0.8 m, constructed from 2 mm thick stainless-steel sheets. The exterior walls were shielded with an asbestos layer. A stainless-steel wire mesh conveyor belt connected the drying chambers and was employed to move samples in and out.

#### Hot air convection system

The convective hot air system has a fan and two electric heaters to generate the required drying air velocity (0.2 to 4 m/s). The airflow into the chamber was controlled by a valve positioned at the inlet of the PVC pipe. The air was warmed by flowing over two spiral electric heaters with a 1.5 kW size. A control unit manages these heaters to ensure a steady temperature throughout the drying process.

#### Infrared heat system

The infrared heating system was equipped with 1000 W halogen lamps (Philips, tube type), each measuring 35.5 cm long and 0.6 cm in diameter. With a 1500–6000 W/m² heating intensity range, the IR lamps were mounted on the drying chamber’s upper interior surface to enable even heating. The IR lamps parallel the conveyor belt at a 15 cm spacing between them. The infrared radiation intensity of the lamps could be varied by regulating the voltage through a power regulator.

#### Conveyor-belt system

A switch could halt the conveyor when the product was placed directly beneath the infrared heaters. This dryer lets infrared and hot air heating be used independently or instantaneously by switching the heating systems on and off. The air temperature during the drying process was tracked with T-type thermocouples linked to a data logger, which has an accuracy of ± 1 °C. In contrast, the air velocity within the chamber was measured using a hot wire anemometer with an accuracy of ± 0.1 m/s.

### Drying technique

The dryer was established at drying airflows of 0.7, 1.0, and 1.5 m/s applied at 40, 50, and 60 °C, drying air temperatures under different infrared intensities (IR) of 1500, 2000, and 3000 W/m². The dryer was run for 45 min to achieve steady-state drying conditions before each drying test. The samples were dried until their final moisture content dropped below 0.05 g water/g dry basis (total drying time ranged from 10 to 5 h). The dried garlic slices were crushed by grinding, and the garlic powder was stored at -20 °C until use.

### Moisture content

About 20 g of the sample was placed in a dish and dried in a vacuum oven at 85 kPa and 70 °C for 24 h^21^ for garlic’s initial moisture content. After drying, the sample was weighed three times to obtain an average value. The garlic’s initial moisture content was about 67.8 ± 0.8% (w.b.). The garlic’s moisture content (*MC*_*db*_) was determined using Eq. 1^22^.

1 $$\:{\text{M}\text{C}}_{db}=\:\frac{{\text{W}}_{i}-{\text{W}}_{\text{d}}}{{\text{W}}_{d}}\:\:\:\:\:\:\:\:\:\:\:\:\:\:\:\:\:\:\:\:\:\:\:\:\:\:\:\:\:\:\:\:\:\:\:\:\:\:\:\:\:\:\:\:\:\:\:\:\:\:\:\:\:\:\:\:\:\:\:\:\:\:\:\:\:\:\:\:\:\:\:\:\:\:\:\:\:\:\:\:\:\:\:\:\:\:\:\:\:\:\:\:\:\:\:\:\:\:\:\:\:\:\:\:\:\:\:\:\:\:\:\:\:\:\:\:\:\:\:\:\:\:\:\:$$

The final moisture content (M_f_) was determined on dry bases following Eq. 2^23^.

2 $$\:{M}_{f}=\:\frac{{W}_{wet}-\:{W}_{d}}{{W}_{d}}\:\:\:\:\:\:\:\:\:\:\:\:\:\:\:\:\:\:\:\:\:\:\:\:\:\:\:\:\:\:\:\:\:\:\:\:\:\:\:\:\:\:\:\:\:\:\:\:\:\:\:\:\:\:\:\:\:\:\:\:\:\:\:\:\:\:\:\:\:\:\:\:\:\:\:\:\:\:\:\:\:\:\:\:\:\:\:\:\:\:\:\:\:\:\:\:\:\:\:\:\:\:\:\:\:\:\:\:\:\:\:\:\:\:\:\:\:\:\:\:\:\:\:\:\:\:\:\:\:$$

The Mt of the dry sample over time t is given by Eq. 3^24^.

3 $$\:{M}_{t}=\:\left[\frac{\left({M}_{i}+1\right)\:{W}_{0}}{{W}_{t}}-1\right]=\frac{{W}_{t}-{W}_{d}}{{W}_{d}}\:\:\:\:\:\:\:\:\:\:\:\:\:\:\:\:\:\:\:\:\:\:\:\:\:\:\:\:\:\:\:\:\:\:\:\:\:\:\:\:\:\:\:\:\:\:\:\:\:\:\:\:\:\:\:\:\:\:\:\:\:\:\:\:\:\:\:\:\:\:\:\:\:\:\:\:\:\:\:\:\:\:\:\:\:\:\:\:\:\:\:\:\:$$

### Water activity (aw)

A 3 g sample was used to measure water activity (a_w_) at 25 °C by the LabMASTER technique. The machine measures the air humidity by closing the samples in a controlled closed chamber to accurately and reliably assess water activity (a_w_).

### Vitamin C content

The vitamin C content of the sample was determined using the 2,6-dichlorophenol indophenol standard titration method^21^. The reagents used were 3% HPO3 (meta-phosphoric acid) solution, ascorbic acid standard containing 0.1 mg Lascorbic acid in 1 ml of 3% HPO3, and the dye solution containing 50 mg of 2,6-dichlorophenol-indophenol in 200 ml of distilled water. The vitamin C retention rate was calculated according to the Eq. 4.

4 $$\:VC=\:\frac{{V}_{t}}{{V}_{o}}\:\:\:\:\:\:\:\:\:\:\:\:\:\:\:\:\:\:\:\:\:\:\:\:\:\:\:\:\:\:\:\:\:\:\:\:\:\:\:\:\:\:\:\:\:\:\:\:\:\:\:\:\:\:\:\:\:\:\:\:\:\:\:\:\:\:\:\:\:\:\:\:\:\:\:\:\:\:\:\:\:\:\:\:\:\:\:\:\:\:\:\:\:\:\:\:\:\:\:\:\:\:\:\:\:\:\:\:\:\:\:\:\:\:\:\:\:\:\:\:\:\:\:\:\:\:\:\:\:\:\:\:\:\:\:\:\:\:\:\:\:\:\:$$

### Flavor strength determination

The Chloramine-T method assessed the volatile oil containing sulfur compounds responsible for garlic’s pungency. The volatile oil content in the material was determined in mg oil per g of dry matter^25^.

### Allicin content determination

The amount of allicin in the initial and dry sample was measured at 412 nm based on the spectrophotometric method detailed by^26^. The allicin content (AC) was extracted as relative retention compared to fresh garlic, as described in Eq. 5.

5 $$\:AC\:\left(\%\right)=\frac{{A}_{t}}{{A}_{o}}\times\:100\:\%\:\:\:\:\:\:\:\:\:\:\:\:\:\:\:\:\:\:\:\:\:\:\:\:\:\:\:\:\:\:\:\:\:\:\:\:\:\:\:\:\:\:\:\:\:\:\:\:\:\:\:\:\:\:\:\:\:\:\:\:\:\:\:\:\:\:\:\:\:\:\:\:\:\:\:\:\:\:\:\:\:\:\:\:\:\:\:\:\:\:\:\:\:\:\:\:\:\:\:\:\:\:\:\:\:\:\:\:\:\:\:\:\:\:\:\:\:\:\:$$

*A*_*o*_ and *A*_*t*_ are the allicin of fresh (mg/g) and dried (mg/g) samples (mg/g), respectively.

### The total color changes measurement (δE)

The color of fresh and dried samples was assessed with a colorimeter according to the method outlined by^27^. In summary, five color capacities were taken from the sample’s surface for each examination, and the mean color changes were verified. The total color changes (δE) were computed using Eq. 6.

6 $$\:\delta\:E=\:\sqrt{{\left({L}^{*}-{L}_{o}^{*}\right)}^{2}+{\left({a}^{*}-{a}_{o}^{*}\right)}^{2}+{\left({b}^{*}-{b}_{o}^{*}\right)}^{2}}\:\:\:\:\:\:\:\:\:\:\:\:\:\:\:\:\:\:\:\:\:\:\:\:\:\:\:\:\:\:\:\:\:\:\:\:\:\:\:\:\:\:\:\:\:\:\:\:\:\:\:\:\:\:\:\:\:\:\:\:\:\:\:\:\:\:\:\:\:\:\:\:$$

L, b, and a denote whiteness, yellowness/blueness, and redness/greenness values. The subscript “0” represents the color value of the garlic. A high value of total color changes (δE) means a greater change in the blanched sample color.

### Machine learning approaches

#### Artificial neural network (ANN)

**Fig. 1:**
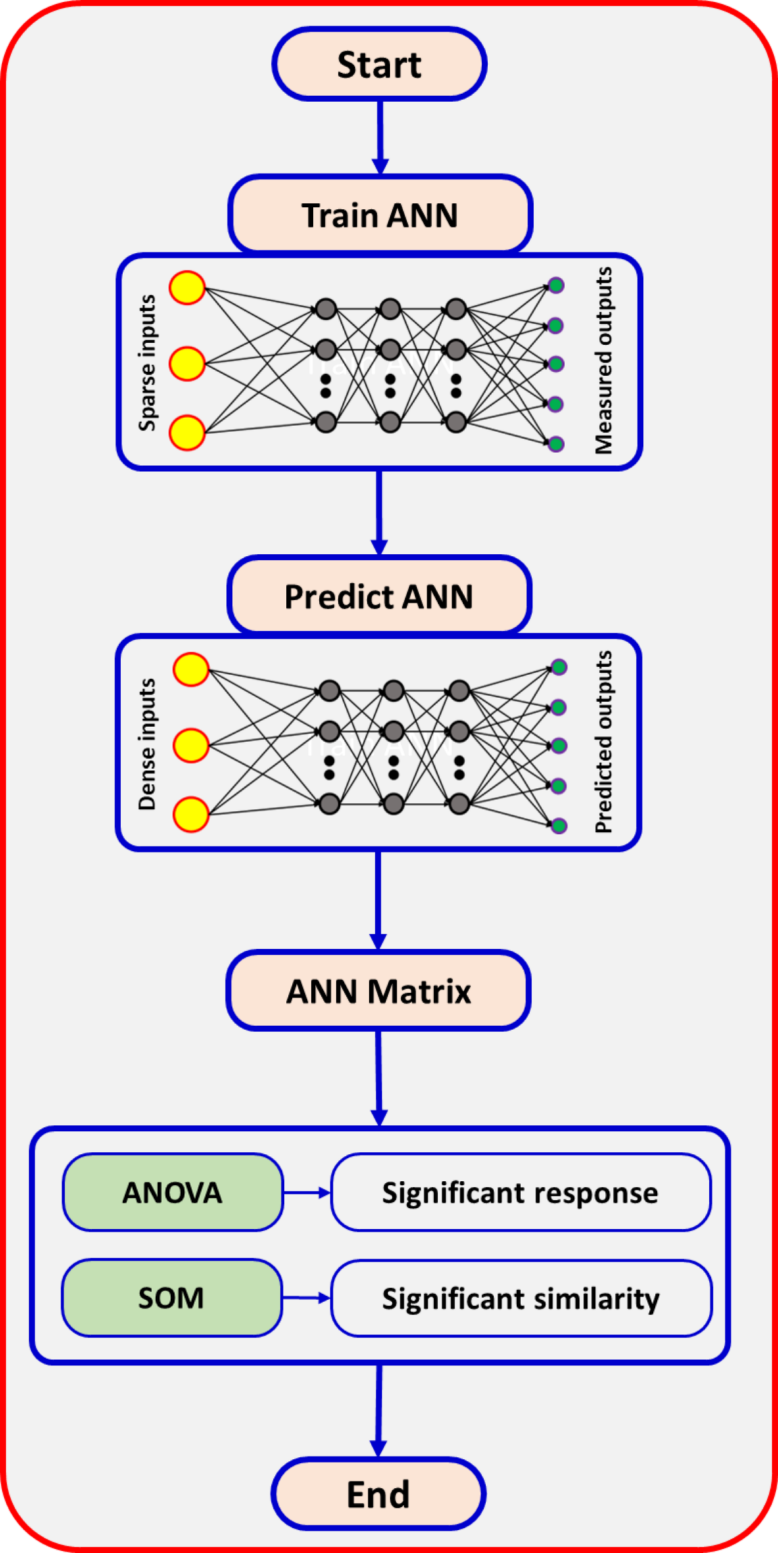
The road map for Artificial Neural Networks (ANNs) is employed in the present investigation.

**Fig. 2:**
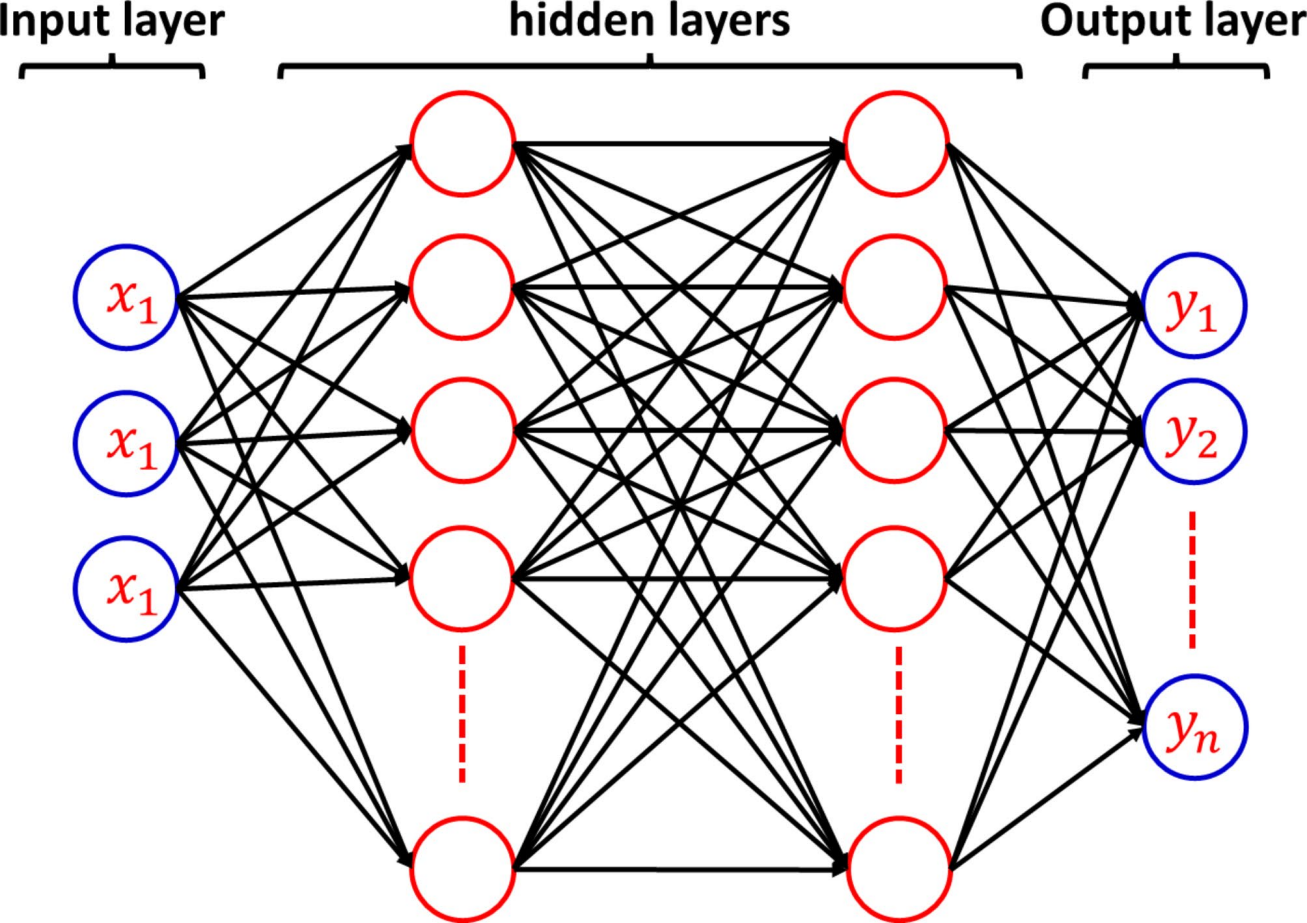
The structure for three layers during training, testing, and validation of the Artificial Neural Networks (ANNs) model.

Artificial Neural Networks (ANNs) are a powerful, data-driven artificial intelligence technique. It is machine learning-based modeling with a supervised learning concept. An ANN consists of three layers: input, hidden, and output. Data is transmitted via the neurons from an input layer to the layers below. The neurons in the output and input layers significantly impact the variables. Although having more neurons can reduce errors, it is also more time-consuming. Weights and transfer functions modify the signals passing through the neurons, and this process repeats until the desired output is achieved. The number of neurons in each layer can vary depending on the problem’s structure^28,29^. Figure 1 illustrates the procedure, including the training and prediction stages of the ANN. The ANN architecture in Fig. 2 includes input, output, and hidden layers. The current ANN model comprises three input factors: air temperature (T), airflow (V), infrared intensity (IR), and garlic physicochemical properties.

The ANN model was successfully integrated with other methods, mainly the (SOM) approach, to optimize drying conditions and provide valuable data on a drying type. The SOM procedure, a key example of unverified machine learning-based modeling with an unsupervised learning concept, organizes varied data into a two-dimensional feature map. According to Ali Hameed et al.^30^, this map maintains the topologic structure of the statistics points via clustering them according to their highest similarity. When these input shapes are presented in the neural network, competition between the output layer parts takes place, resulting in the activation of ‘winning’ neurons. This concept, where weights closely match the input pattern, is better explained in the study conducted by Kaveh et al.^31^ to identify the winner. The above presents the architecture of the network: a single layer applies these conditions to the neurons, which then successfully combine to form n × m clusters. The system processes different parameters: total color change, flavor strength, water activity, vitamin C, and allicin content. Organizing these output parameters into 20 × 20 clusters resulted in 400 output neurons. Clustering was successfully executed using a method comparable to analysis. This approach facilitates the creation of well-organized and understandable data groupings.

#### Principal component analysis (PCA)

**Fig. 3:**
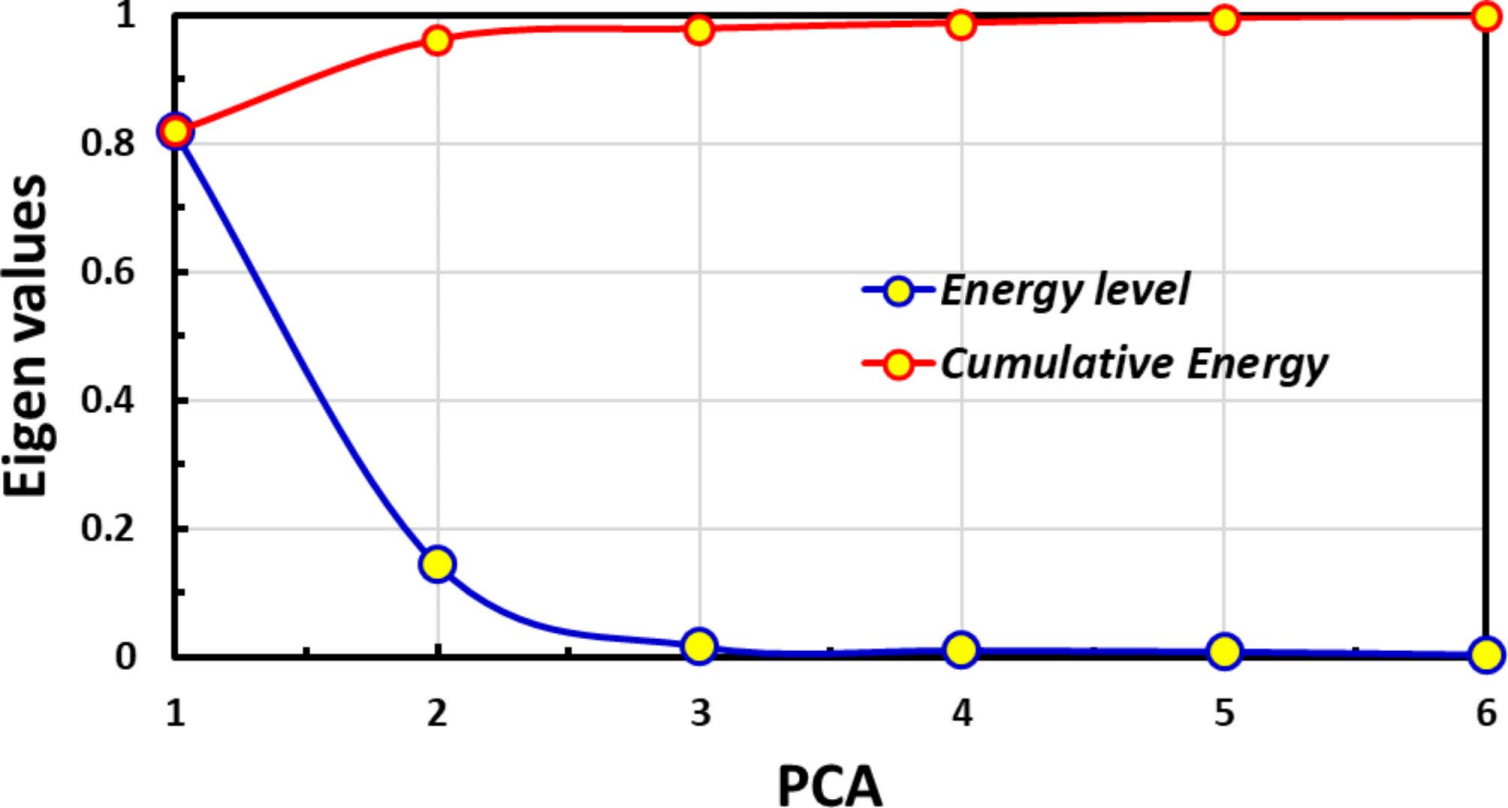
Principal component groups of the predicted quality contents using eigenvalues.

Principal Component Analysis (PCA), carried out with the Minitab 2022 LLC software, allowed for adding statistical details. This statistical technique tried deciphering the complex relations and differences between garlic’s physicochemical properties. The eigendecomposition of the covariance matrix incorporates the anticipated data sets (total color change, flavor strength, water activity, vitamin C, and allicin concentration) from the artificial neural network (ANN). The first two mechanisms were selected based on total variance and eigenvalue, crucial factors in choosing and explaining Principal Component Analysis (PCA)^32^. Figure 3 shows the PCA analysis of the governing parameters, identifying six components. Considering Kaiser-Guttman criteria, only eigenvalues higher than unity are considered, and thus, only the first two principal components, PC1 and PC2, are utilized to showcase the relationships among the various quality of garlic.

### Validation methodology

**Fig. 4:**
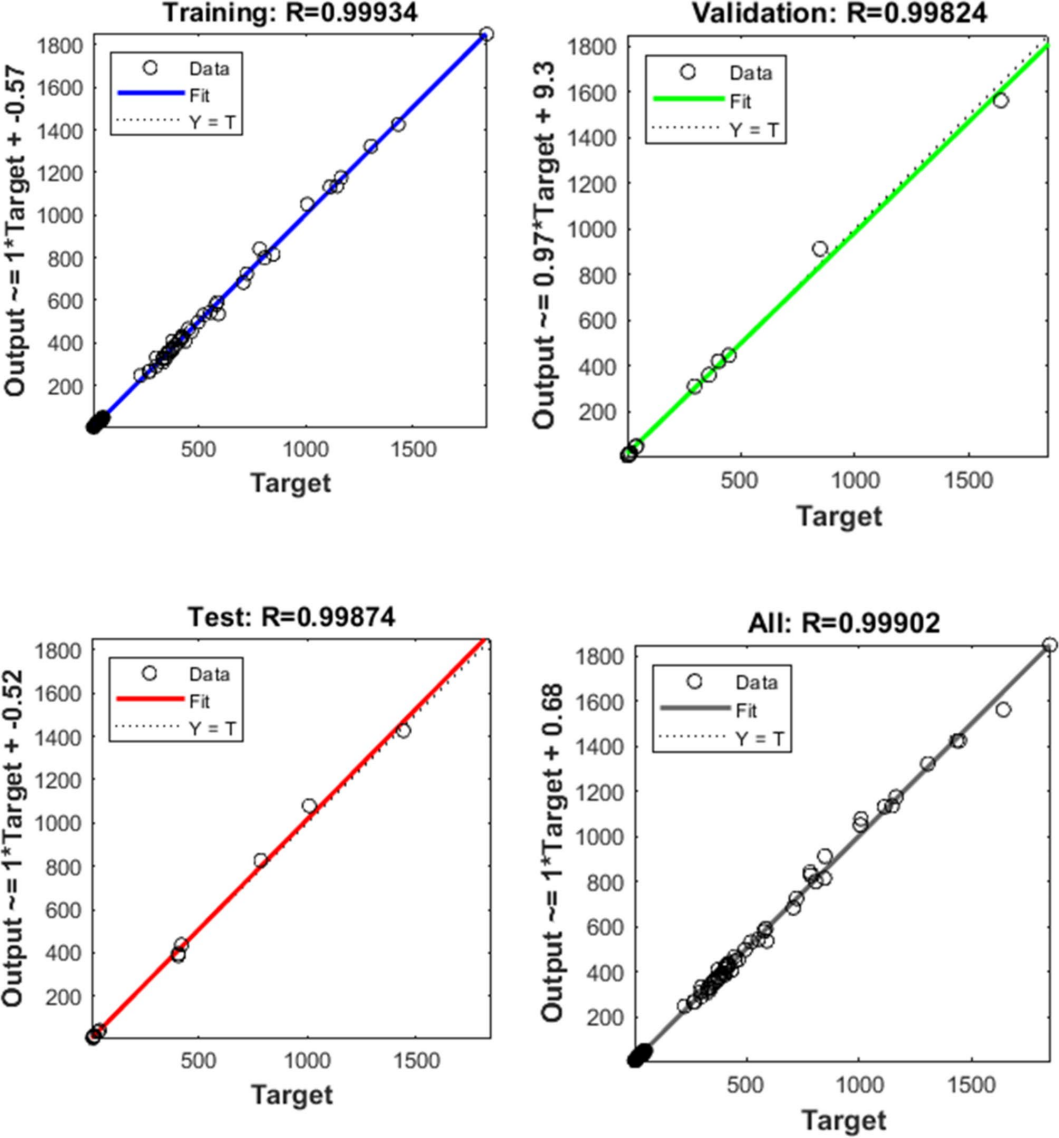
The regression performance during training, testing, and validation of the ANN model.

**Fig. 5:**
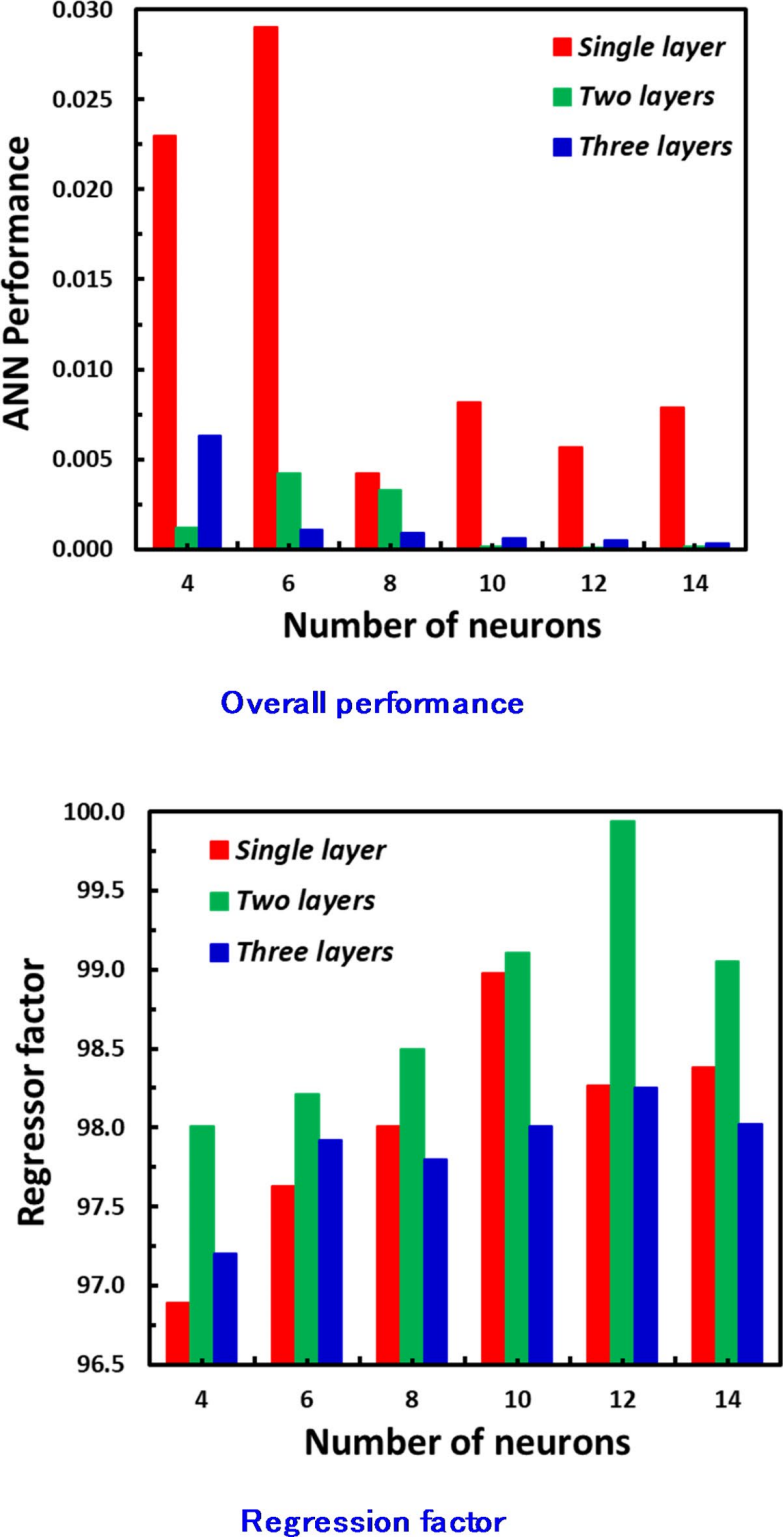
The ANN’s functioning and regression with hidden layers and neurons.

The back-propagation technique, utilizing the sigmoid function, is employed during the ANN training stage. For the ANN model, private code was developed using MATLAB software. The dataset was randomly split, with 70% allocated for training and 30% for testing. The ANN setup featuring 12 neurons across two hidden layers offered the optimal equilibrium among functioning and regression, as illustrated in Fig. 4. Figure 5 illustrates the ANN’s regression and functioning by 1 to 3 hidden layers and 4 to 14 neurons with 99% predicting accuracy. Back-propagation is a training method for ANN that remains stable at low learning rates. Additionally, all cases are subjected to the sigmoid function, as described in Eq. 7:

7 $$\:f\left(x\right)=\frac{1}{1+{e}^{-x}}\:\:\:\:\:\:\:\:\:\:\:\:\:\:\:\:\:\:\:\:\:\:\:\:\:\:\:\:\:\:\:\:\:\:\:\:\:\:\:\:\:\:\:\:\:\:\:\:\:\:\:\:\:\:\:\:\:\:\:\:\:\:\:\:\:\:\:\:\:\:\:\:\:\:\:\:\:\:\:\:\:\:\:\:\:\:\:\:\:\:\:\:\:\:\:\:\:\:\:\:\:\:\:\:\:\:\:\:\:\:\:\:\:\:\:\:\:\:\:\:\:\:\:\:\:\:\:\:\:$$

Before being integrated into the ANN framework, the data set was adjusted according to Eq. 8:

8 $$\:{X}_{i}=\frac{{x}_{i,max}-{x}_{i}}{{x}_{i,max}-{x}_{i,min}}\:\:\:\:\:\:\:\:\:\:\:\:\:\:\:\:\:\:\:\:\:\:\:\:\:\:\:\:\:\:\:\:\:\:\:\:\:\:\:\:\:\:\:\:\:\:\:\:\:\:\:\:\:\:\:\:\:\:\:\:\:\:\:\:\:\:\:\:\:\:\:\:\:\:\:\:\:\:\:\:\:\:\:\:\:\:\:\:\:\:\:\:\:\:\:\:\:\:\:\:\:\:\:\:\:\:\:\:\:\:\:\:\:\:\:\:\:\:\:\:\:\:$$

### Evaluation of model performances

The technique known as Levenberg-Marquardt was used to minimize errors, and after 1000 repeats, the minimum error was recorded. Statistical measures such as correlation coefficient (R²) and root mean square error (RMSE) were operated to estimate the performance of the models. The correlation coefficient (R²) and mean square error (RMSE) are described in Eqs. 9 and 10^33^.

9 $$\:{R}^{2}=\frac{\sum\:_{i=1}^{N}{\left({MR}_{exp.i}-{MR}_{Pre.i}\right)}^{2}}{\sqrt{\left[\sum\:_{i=1}^{N}{\left({MR}_{exp.i}-{MR}_{Pre.i}\right)}^{2}\right]*\left[\sum\:_{i=1}^{N}{\left({MR}_{exp.i}-{MR}_{Pre.i}\right)}^{2}\right]}}\:\:\:\:\:\:\:\:\:\:\:\:\:\:\:\:\:\:\:\:\:\:\:\:\:\:\:\:\:\:\:\:\:\:\:\:\:\:\:\:\:\:\:\:\:\:\:\:\:\:$$

10 $$\:RMSE=\sqrt{\frac{1}{n}\sum\:_{i=1}^{n}{\left({X}_{m}-{X}_{p}\right)}^{2}}\:\:\:\:\:\:\:\:\:\:\:\:\:\:\:\:\:\:\:\:\:\:\:\:\:\:\:\:\:\:\:\:\:\:\:\:\:\:\:\:\:\:\:\:\:\:\:\:\:\:\:\:\:\:\:\:\:\:\:\:\:\:\:\:\:\:\:\:\:\:\:\:\:\:\:\:\:\:\:\:\:\:\:\:\:\:\:\:\:\:\:\:\:\:\:\:\:\:\:\:\:\:\:\:\:\:$$

Triplicate experiments were performed for each drying condition. The experimental data are presented as means ± standard deviations (SD). Statistical analyses were performed using SPSS statistics software (version 27.0, SPSS Inc., Chicago, IL, USA). The effects of various operating settings on the drying properties and quality parameters were assessed using an analysis of variance (ANOVA), which proceeded by a post hoc Duncan’s multiple range test at a significance rank of 0.05.

## Results and discussion

### Water activity (aw)

**Fig. 6:**
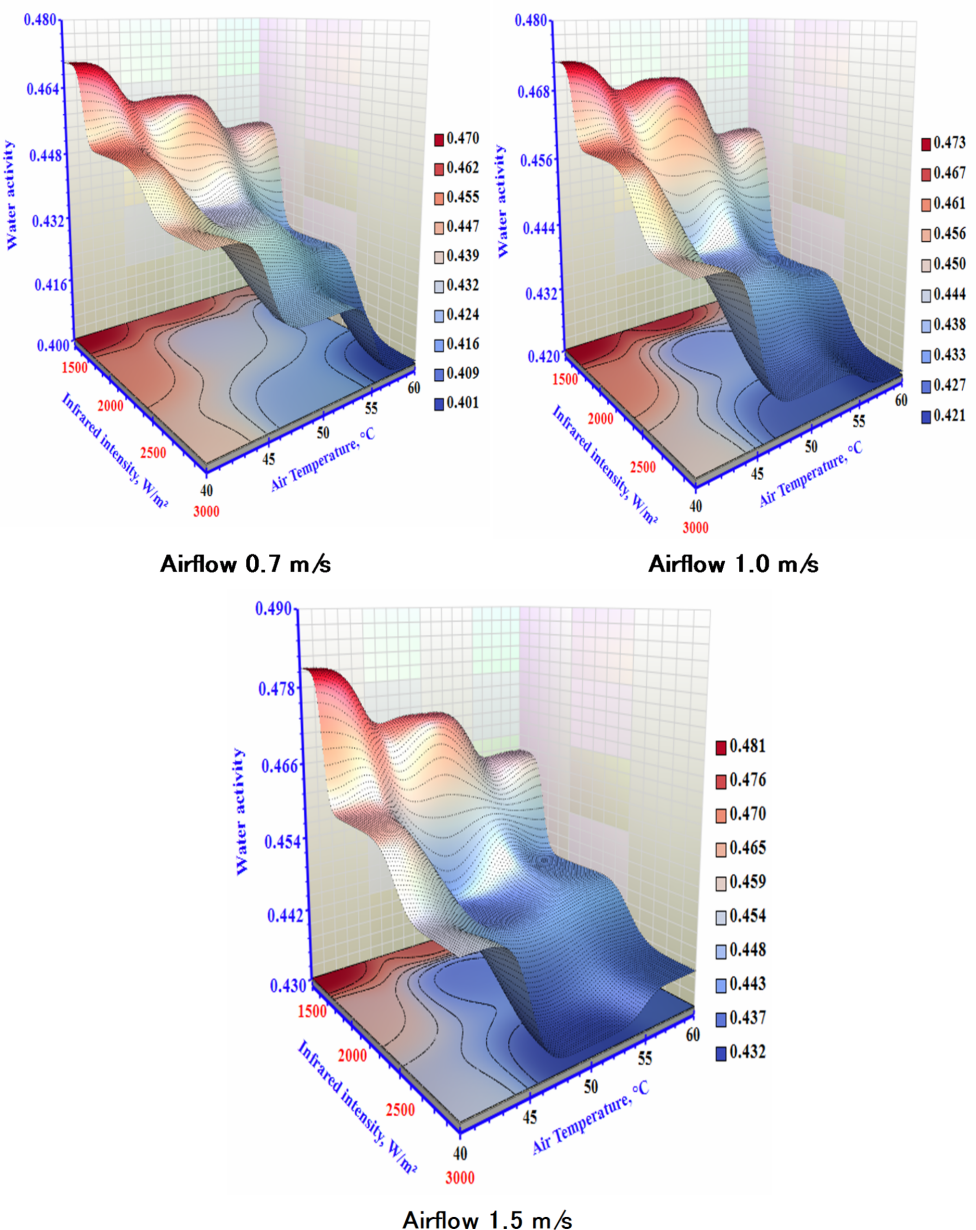
The impact of infrared power and air temperature on the water activity of dried garlic at different air airflows.

A dietary substance’s water activity (aw) indicates that low water activity food has a water activity level of less than 0.8. This meant the required water activity for dry products was 0.6, which is the standard limitation for reproducing yeast and microbes^34^. Figure 6 displays the (a_w_) of the sample as a function of different drying settings. The (a_w)_ of the sample is lower than 0.6 in all tests, signifying that the samples are free from archetypal bacterial harm. The (a_w_) values for the sample ranged from 0.43 to 0.48. Rising IR and T with dropping airflow is the reason for the faster moisture evaporation from sample^35^. Faster achievement of the required (a_w_) is made possible by more forceful water loss from the slice and more active diffusion of moisture inside the sample^36,37^. The findings of this investigation show that drying using an infrared heating process can offer reliable (a_w_) values for extended shelf life for the garlic.

### Vitamin C content

**Fig. 7:**
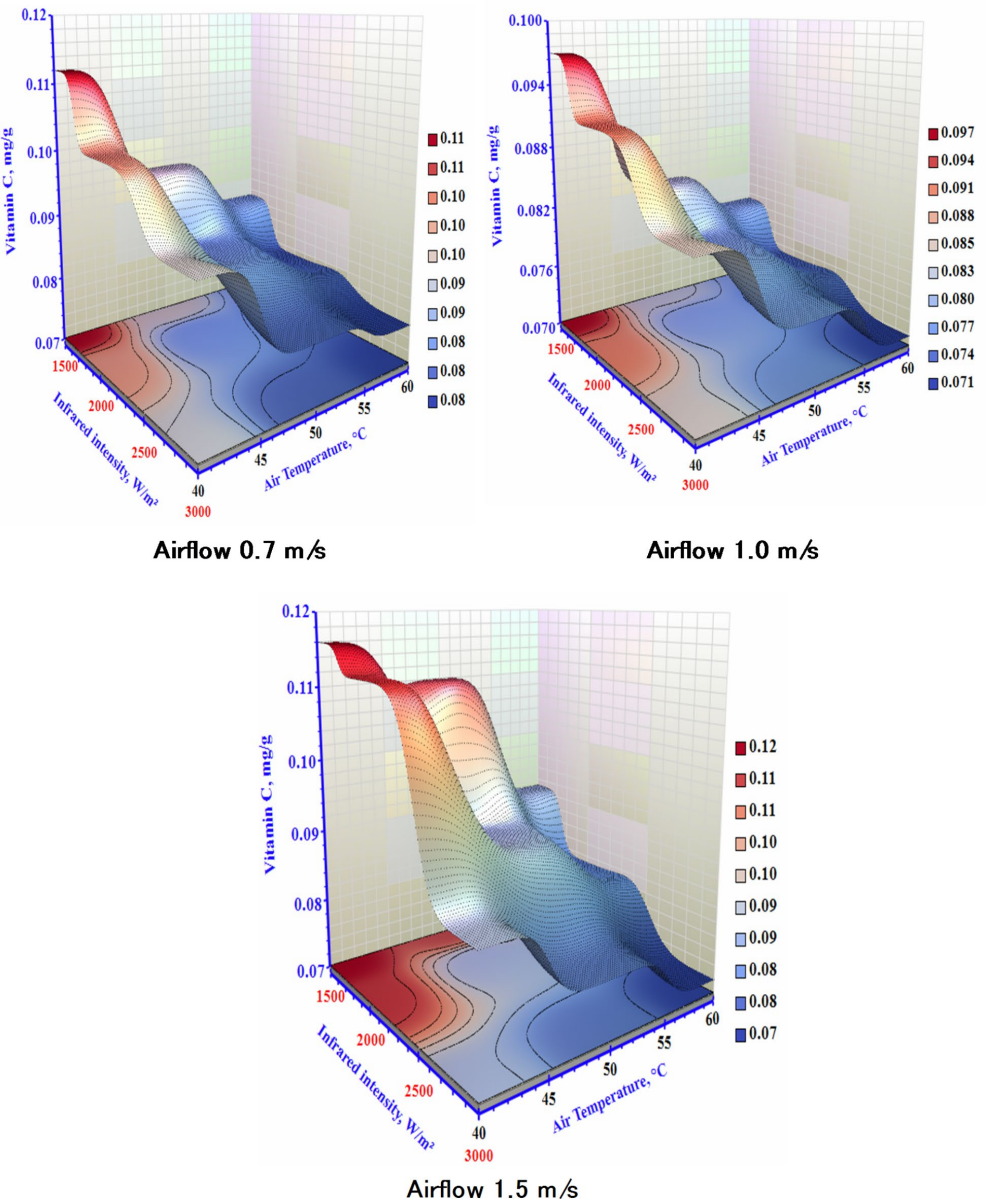
The impact of infrared power and air temperature on the vitamin C content of dried garlic at different air airflows.

Vitamin C can be found in most fresh fruits and vegetables; however, it is not very stable and is usually degraded during processing when subjected to heat, oxygen, or light^38^. The influence of different air temperatures, airflow, and IR on the Vitamin C of the dried sample is shown in Fig. 7. The vitamin C content of dried garlic decreased with an increase in air temperature and radiation intensity. The minimum value of vitamin C content was observed at 60°C, and 1.5 m/s under 3000 W/m² was 0.05 mg/g. The maximum vitamin C content was 0.112 mg/g, followed by an air temperature of 40°C and 0.7 m/s air velocity under 1500 W/m². Due to its heat sensitivity, vitamin C quickly degrade. A short drying time using low air temperature methods is advised to preserve vitamin C while attaining significant dry inefficiencies. It will break down by increasing the duration of the dehydrating process. At higher levels of T and IR, the lowest level of vitamin C was recorded. The samples’ additional time in the dryer and higher IR intensity, which ultimately changed the composition of the finished good and caused thermal degradation, is responsible for the drop in vitamin C^39^. The improvement of the conditions for drying garlic using microwave was examined by Sharma and Prasad^40^. Their hybrid dryer research discovered that extending the airflow from 1.0 to 2.0 m/s while maintaining the same T and power level lengthened the dehydrating time. In contrast to what might be anticipated during hot air drying, the drying time was increased rather than decreased. One possible explanation is that the material cooled due to the increased air velocity, reducing the sample’ temperature. Nevertheless, with an airflow of 2.0 m/s, 40 W, and 60 °C, the dried sample showed the maximum vitamin C during the drying procedure.

### Allicin content

**Fig. 8:**
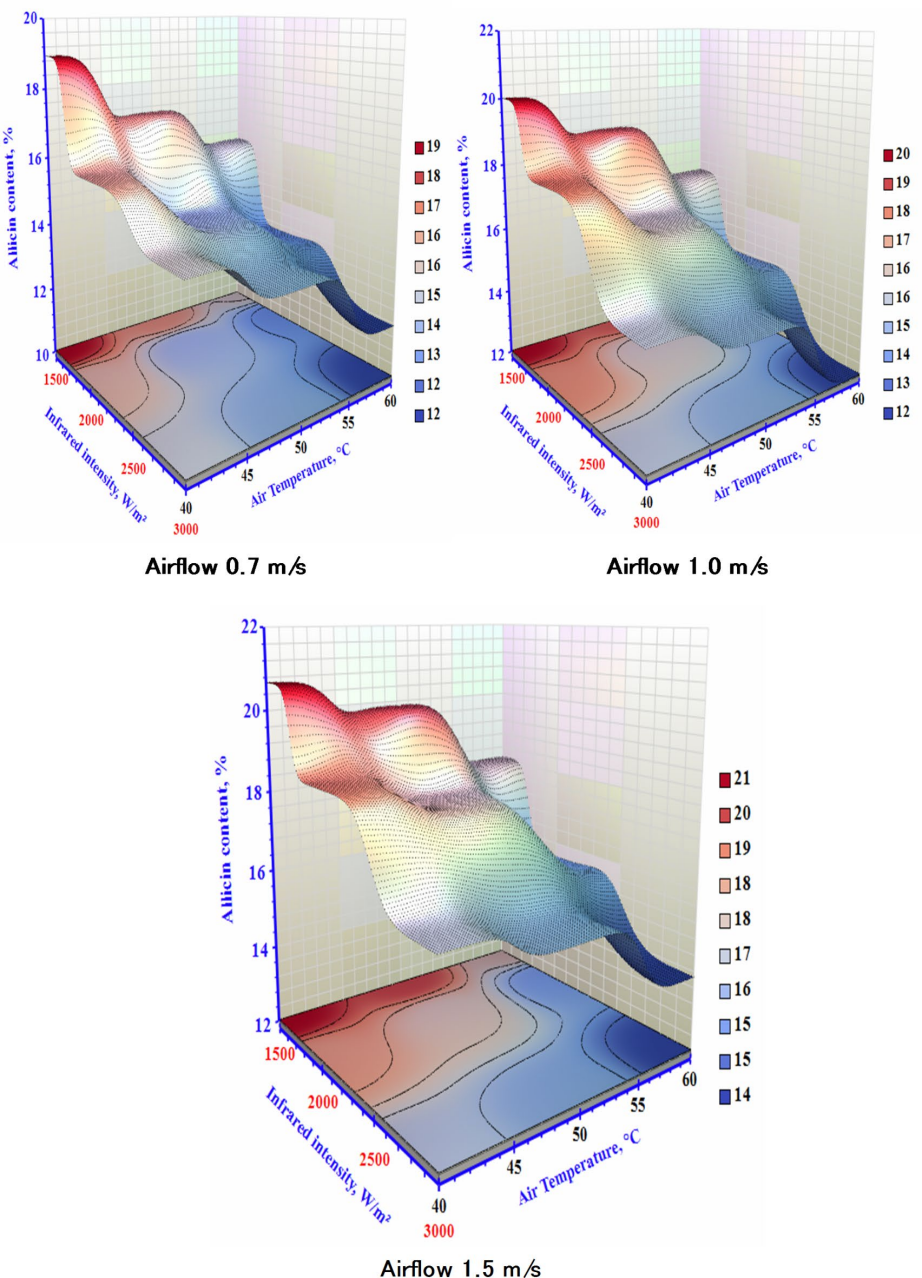
The impact of infrared power and air temperature on the allicin content of dried garlic at different air airflows.

The influence of different T, V, and IR on the allicin content of the dried sample is depicted in Fig. 8. Additionally, there was a noticeable decrease in allicin content with higher levels of IR as the temperature increased, regardless of the velocity rates. This finding can be attributed to the fact that the optimal temperature for alliinase in garlic is 40 °C, and alliinase activity decreases at temperatures above this optimal point^41^. The allicin could be retained better at lower temperatures after the rupture of the garlic cell. This decrease can be attributed to the temperature-induced degradation of alliin, the precursor compound for allicin, as documented by Feng et al.^42^. The reduced moisture content can lead to higher allicin levels, as the rising temperature at 40 and 60 °C can denature and inactivate alliinase in garlic, thereby producing more allicin during the drying process^43^. Usually, higher internal product temperatures speed up allicin’s degradation rate. However, higher IR power levels led to shorter drying times. The balance between these factors produced similar allicin content values in samples dried at varying IR power levels. The comparison of different drying conditions also showed a significant difference, as temperature is the main effect. The substantial increase in allicin content could be linked to the rise in heat-sensitive compounds, with higher temperatures enhancing the conversion of allyl compounds into diallyl thiosulfinates, as reported by Puranik et al.^44^. The allicin content increases with hot air drying as the temperature rises from 40 °C to 70 °C. However, temperatures above 80 °C can cause allicin to break down, resulting in lower content. The airflow significantly impacted the allicin content in the sample. Rising the (V) to 1.5 m/s at the lowest (T) and IR resulted in roughly 55% more allicin paralleled to the lowly allicin content recorded at high (T) and IR levels and low (V).

### Flavor strength

**Fig. 9:**
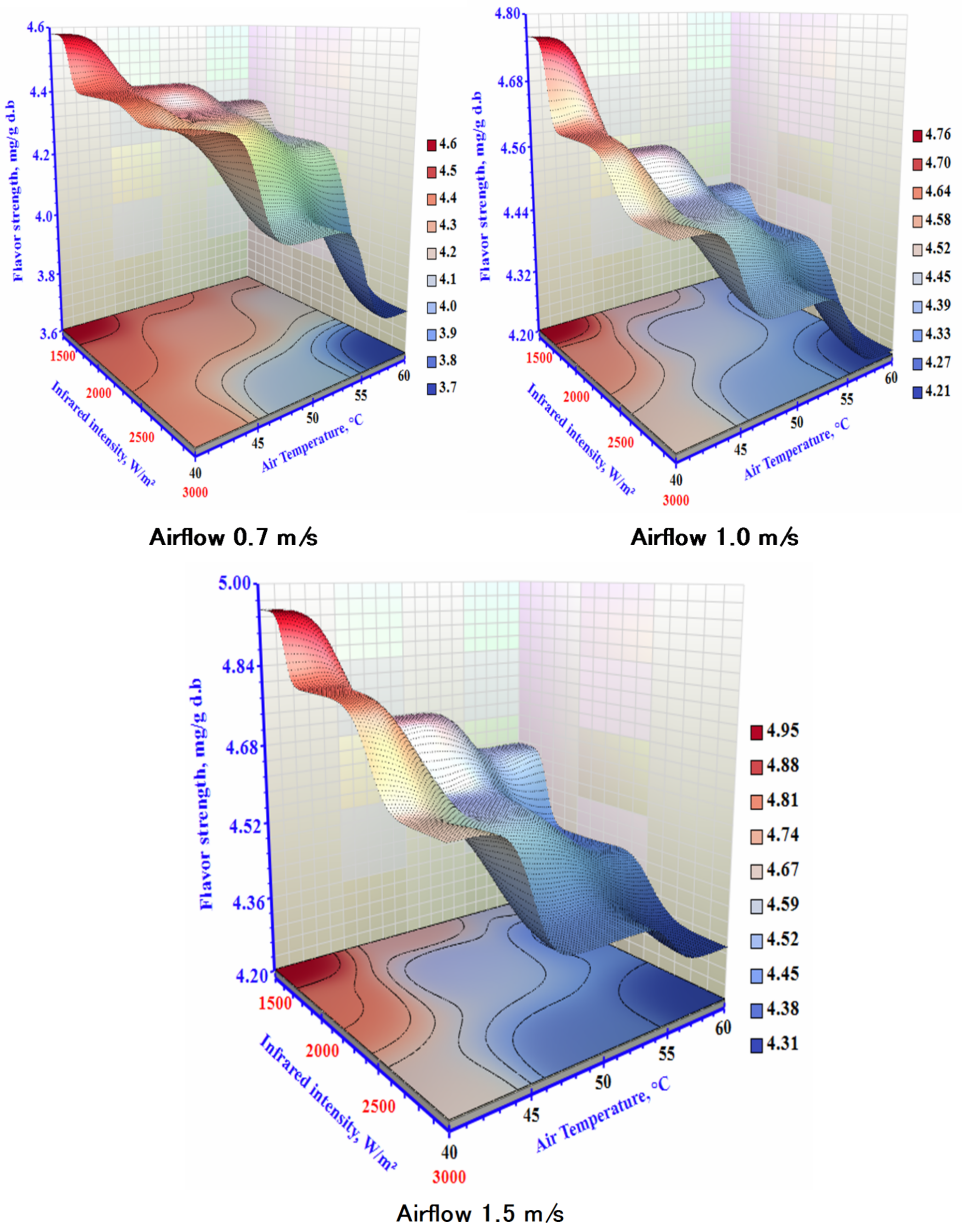
The impact of infrared power and air temperature on the flavor strength of dried garlic at different air airflows.

Considering the perspective of market approval, flavor strength is a crucial quality factor for dry garlic products. Figure 9 displays dried garlic with varied flavor levels across different drying settings. Therefore, the highest flavor content was recorded under high V, low T, and IR settings. Conversely, the lowest flavor level was observed at 60 °C, 3000 W/m², and 0.7 m/s. A flavor decrease was correlated with a drying temperature rise from 40 °C to 60 °C. On the other hand, there was a negative correlation between the airflow and the garlic flavor. Similar outcomes were reported by Feng et al.^26^ for garlic using catalytic infrared drying, Figiel^45^ for vacuum-microwave drying garlic, and Rao et al.^46^, who described a decrease in flavor in the material.

### The total change of color changes

**Fig. 10:**
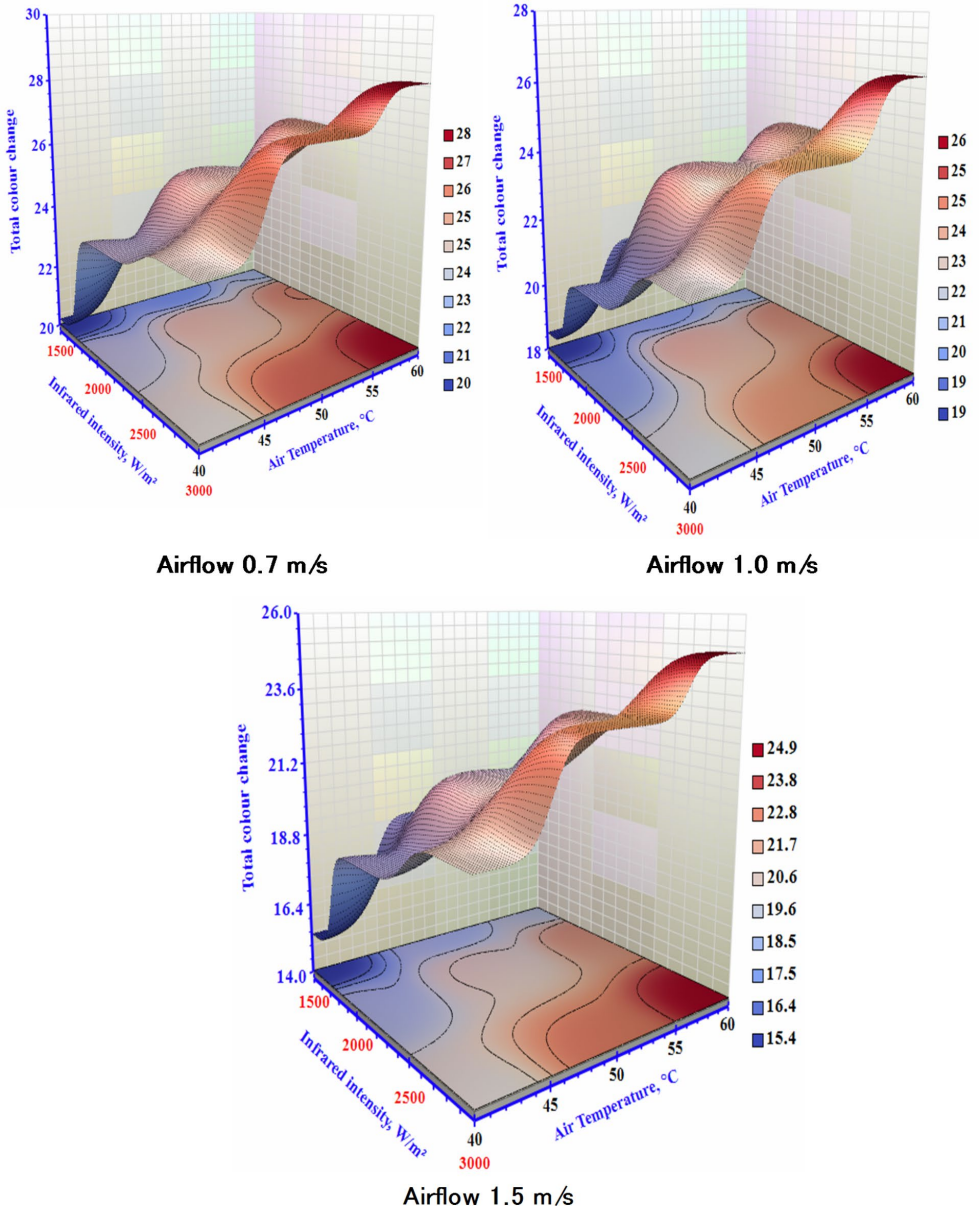
The impact of infrared power and air temperature on the total change of dried garlic at different air airflows.

Color is a crucial quality attribute affecting consumer acceptance and foodstuffs’ market value. The lowest δE is used as the manufacturing standard for assessing the color of dry garlic. The results of the total color change are represented in Fig. 10. The performed statistical analysis indicated that drying air temperature and IR significantly (*P* < 0.05) affected the δE of the dried sample, with δE increasing notably as temperature and IR levels rose. As the drying temperature increased, the δE increased in each treatment due to more severe non-enzymatic browning at higher temperatures^47^. This finding aligns with^48^, who found that extended dehydration processes lead to more pronounced browning reactions, affecting the compounds in the samples and likely influencing pigment degradation and both enzymatic and non-enzymatic reactions. As shown in Fig. 10, the δE increased with higher (IR) but decreased with greater (V). The decrease in δE with increased (V) can be credited to the cooling influence of the passing airflow on the samples within the dehydrating room, resulting in overall heat loss to the chamber^49^. Taghinezhad et al.^50^ investigated the overall color change in organic blackberries during hybrid hot air/infrared drying, both with and without ultrasonic pretreatment, for 15, 30, and 45 min. Their results showed a consistent decrease in the total color variation of the samples as the drying air temperature increased from 50 to 60 °C and from 60 to 70 °C. Similarly, Özbek^51^ examined the overall sweet potatoes color change using hybrid infrared/hot air drying. The study found that higher air temperatures led to smaller changes in the color of samples with a thickness of 6 mm.

### Discrimination performance results

**Fig. 11:**
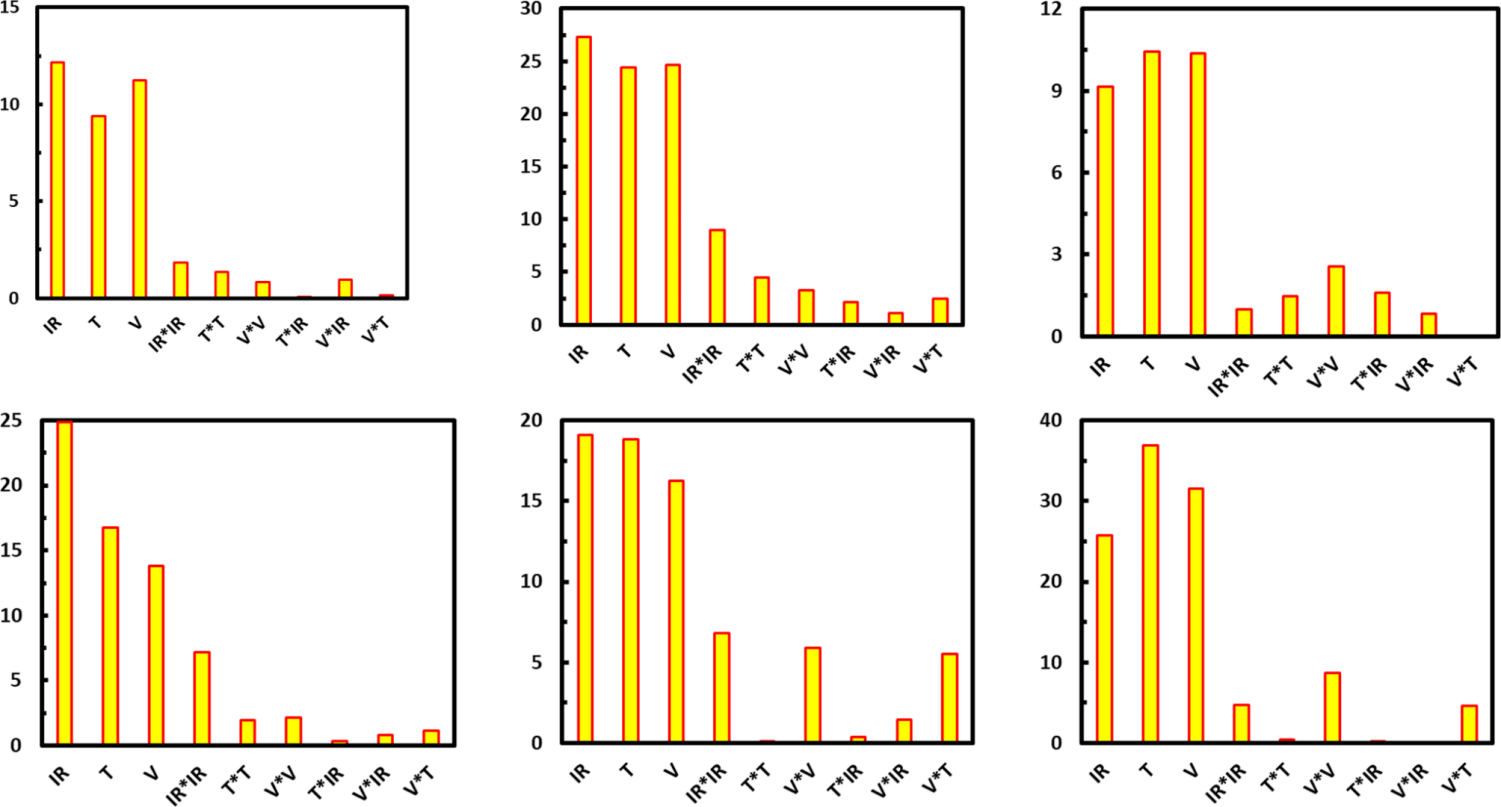
Pareto charts for physicochemical properties of dried garlic using an infrared heating system under different drying conditions.

**Fig. 12:**
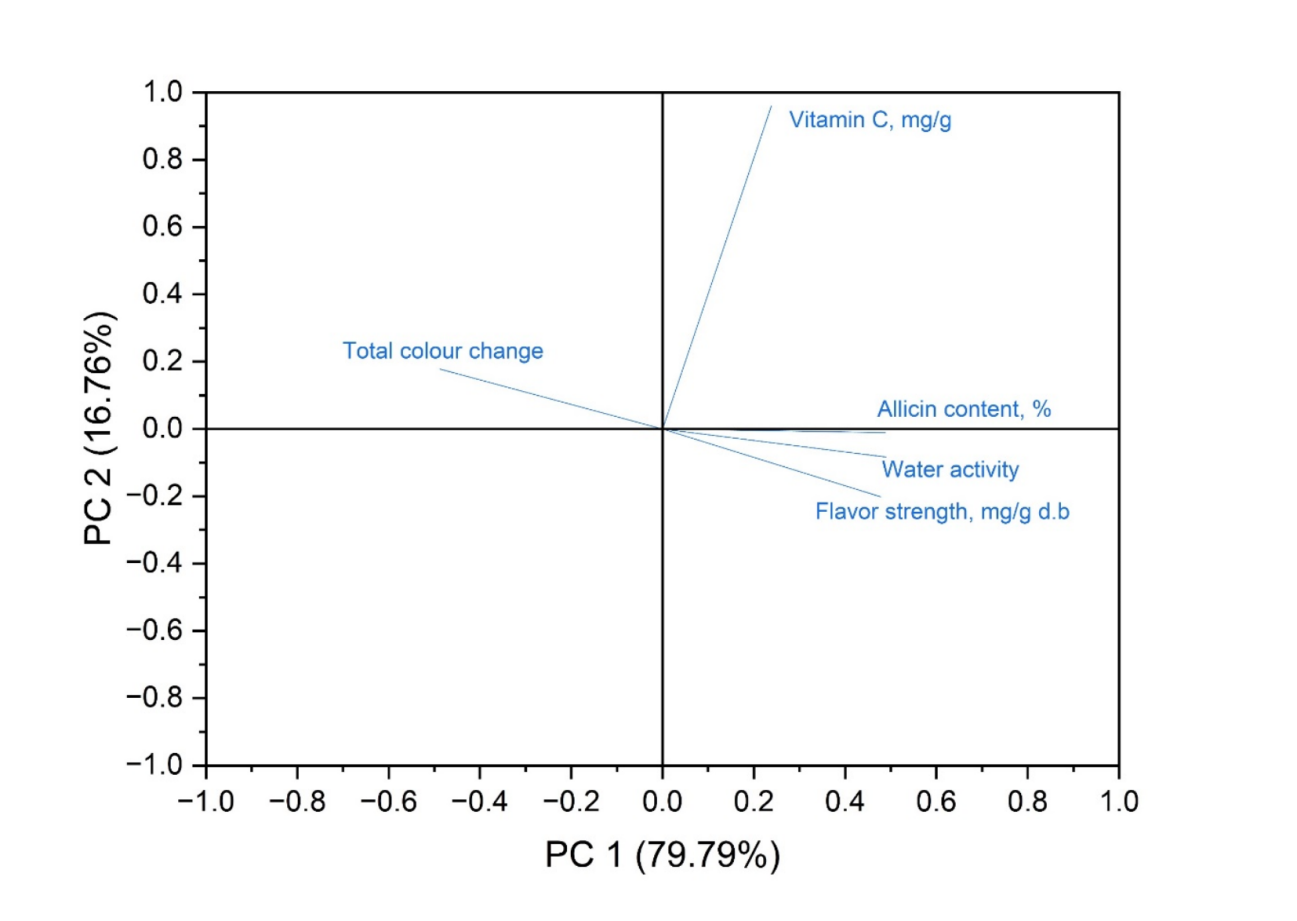
Principal component analysis of dried garlic slices subjected to infrared power, airflow, and drying air temperature.

In many different configurations, the Artificial Neural Network (ANN) is the ideal method for predicting the results of nonlinear structures. Estimating the output data of the intended collection of parameter combinations, overlaying the technique behavior, and filling the network with empirical data are the key components of the procedure^52^. From the Pareto chart (Fig. 11), the limit value was estimated to be 2.11, where the variable would be significant above it^53^ and unimportant below it^54^. Radiation, velocity, and temperature play essential roles in controlling the quality attributes of all variables. Radiation was the first factor influencing color, allicin, and water activity, followed by temperature and velocity. As for flavor and vitamin C, the temperature comes first due to velocity and radiation intensity. The interaction between factors is considered, whereas a single-factor experiment cannot identify interactions amongst the parameters, especially for the color. The velocity contact is more significant for flavor and vitamin C, but the radiation interaction is more important for allicin and water activity. According to the eigenvectors, each variable affects PC1’s structure reasonably, with the vitamin C variable having the most significant impact on all of the parameters in this component’s composition. On the other hand, the most significant variables in PC2 are flavor, water activity, and allicin content; these variables influence the variance and significantly contribute to the differences represented by this main component. Figure 12 shows the relationship between PC1 & PC2 to demonstrate the correlated variables better. The Vitamin C, allicin, and water activity correlate positively, indicating its increment. The total color change, Positive PC1, and negative PC2 are present in the flavor with growing priority, with PC1 preceding. Accordingly, the accompanying information shows that while drying harms color, it has a good impact on flavor, water activity, vitamin C, and allicin.

**Fig. 13:**
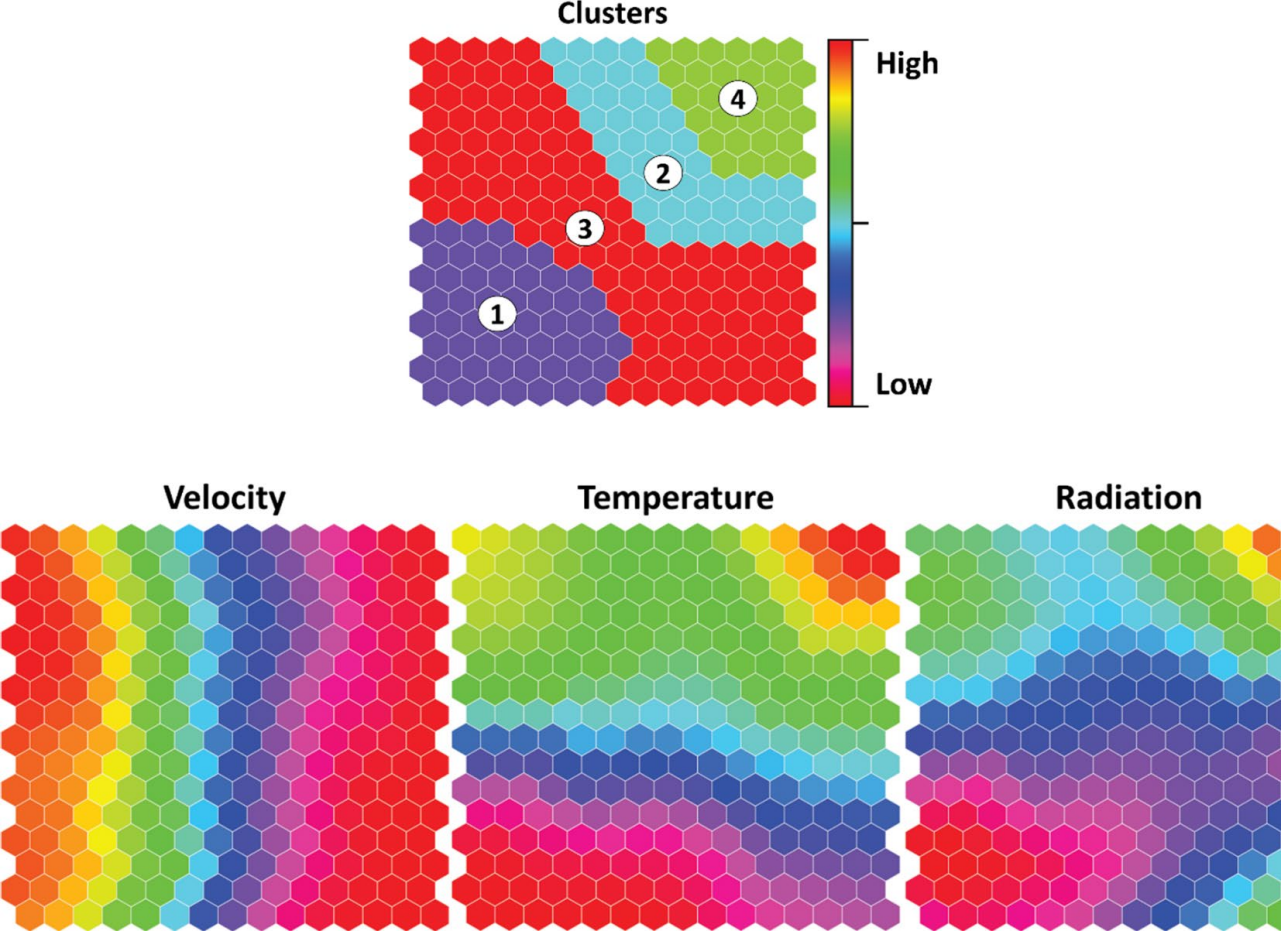
Self-organizing map (SOM) clusters of the matrix for drying conditions.

**Fig. 14:**
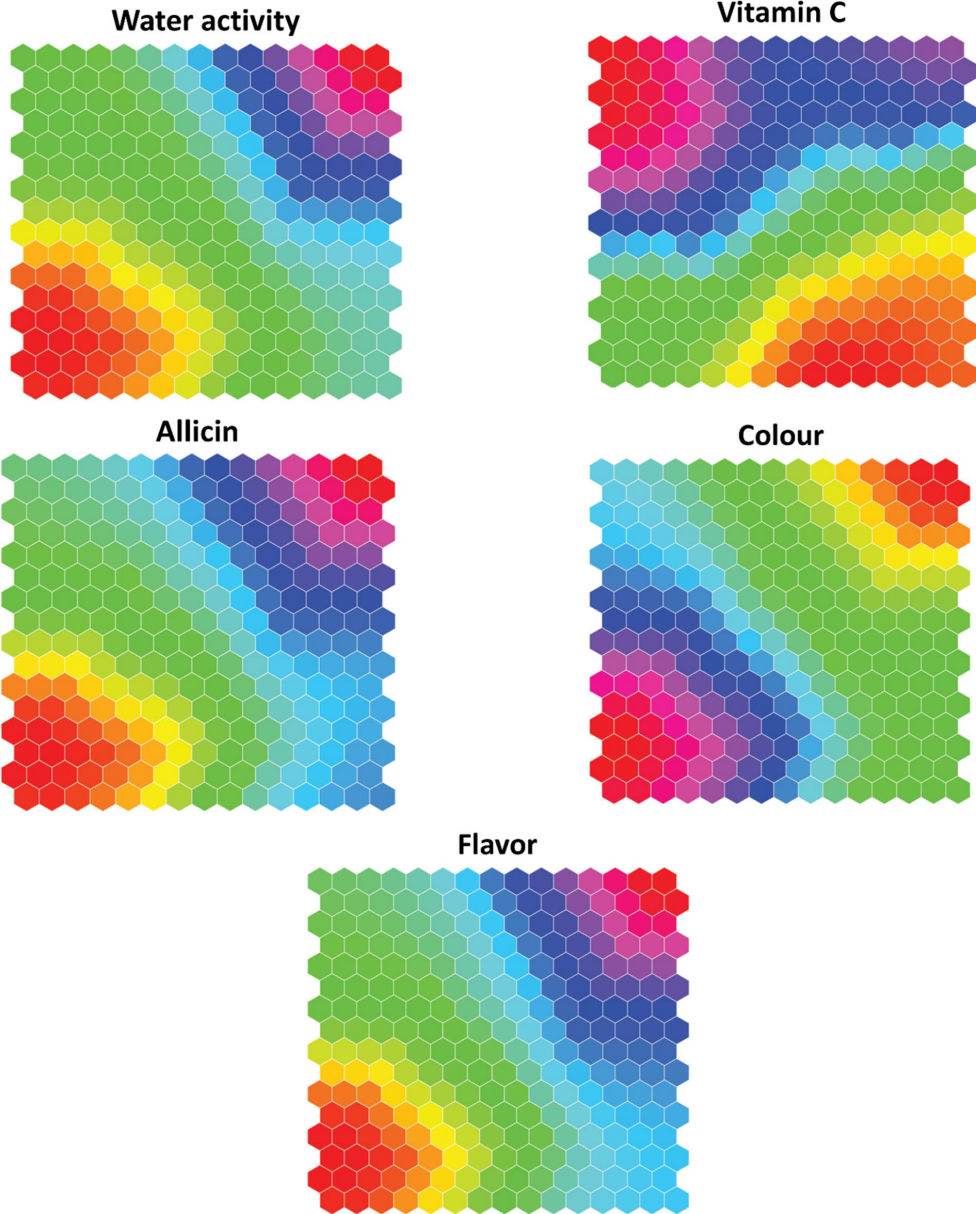
Self-organizing maps clusters of output variables (total color change, flavor strength, water activity, vitamin C, and allicin content) for dried garlic slices.

The ANN simulations are combined using extra algorithms to improve dryer settings or monitor the dehydrating technique through the self-organizing map (SOM) method. Although the previous evaluation provides an extensive overview of the quality material, self-organizing maps (SOM), an exciting information extraction approach, can provide an alternate knowledge of the best working conditions. The SOM representations are shown in Fig. 13, which are grouped into four clusters according to feature similarity, with 97% clustering accuracy. Significant differences in V, T, and IR across all clusters can be observed on the map in Fig. 13, which provides substantial context for our study. The quality characteristics show a considerable variation with clusters in Fig. 14. The map displays high water activity, flavor, color, and medium vitamin C for the first cluster. This cluster has higher values of both convection and radiation. The colour indicates relatively low-to-medium values, while the vitamin C shows medium-to-high values. This cluster has a wide range of convection and radiation changes, from low to high values. The second cluster’s water activity, allicin, and flavor exhibit lower values, while the color shows medium values. This cluster has high-velocity values with medium values for both temperature and radiation. In the fourth cluster, all variables show higher values, whereas both convection and radiation also have higher values.

## Conclusion

The application of machine learning algorithms to improve food drying processes is increasingly popular in the food industry due to their ability to enhance drying settings while maintaining physicochemical properties (total color change, flavor strength, water activity, vitamin C, and allicin content). Artificial Neural Network (ANN) with 99% predicting accuracy and Self-Organizing Maps (SOM) with 97% clustering accuracy were used to determine the quality characteristics of garlic. This investigation examines how various airflow, temperature, and IR affect the physicochemical properties of garlic. The study found that high airflow, low temperature, and IR levels achieved the highest flavor content. In contrast, the lowest flavor content occurred at 0.7 m/s, 60 °C, and 3000 W/m² IR. The total color difference increased with higher IR intensity and air temperature but decreased with lower airflow. Five improvement areas were found using the (SOM) approach; the fourth group indicated the potential for improving quality by keeping constant air temperature and IR. The study highlights the importance of combining advanced statistical analysis and machine learning to optimize foodstuff processing. Future research could investigate the application of these methods across different foods and dehydrating methods, possibly transforming the industry by using statistics to improve product quality and operational efficiency.

Here, we give the following development suggestions to provide some directions for the future drying of food: (a) The development of relevant observation technologies for fruit and vegetable drying, such as computer vision and sensor technology, can enable us to observe the complex process of food drying better. (b) Drying technology based on efficient physical fields can solve many problems in drying fruits and vegetables, but each technology has disadvantages. Different high-efficiency physical field drying technologies can solve different problems. Ultrasonic pretreatment and microwave technologies can increase the drying rate, but the drying uniformity is not good enough. The combination of these efficient physical field drying technologies can be used in different spaces or times to maximize the optimization of dried products. Using the advantages of different artificial intelligence technologies, we can better observe the drying process, control the reasonable application of these efficient physical field drying technologies in time and space, and analyze and model the drying process.

## Data Availability

Data Availability Statement: The original contributions presented in the study are included in the article; further inquiries can be directed to the first author (Hany S. El-Mesery, elmesiry@ujs.edu.cn) and the corresponding author.
